# Dental students’ learning attitudes and perceptions of YouTube as a lecture video hosting platform in a flipped classroom in Korea

**DOI:** 10.3352/jeehp.2018.15.24

**Published:** 2018-10-11

**Authors:** Chang Wan Seo, A Ra Cho, Jung Chul Park, Hag Yeon Cho, Sun Kim

**Affiliations:** 1 Department of Periodontology, Dankook University College of Dentistry, Cheonan, Korea; 2 Department of Medical Education, College of Medicine, The Catholic University of Korea, Seoul, Korea; Hallym University, Korea

**Keywords:** Flipped learning, Dental education, Educational technology, Smartphone, Republic of Korea

## Abstract

**Purpose:**

The aim of this study was to confirm the applicability of YouTube as a delivery platform of lecture videos for dental students and to assess their learning attitudes towards the flipped classroom model.

**Methods:**

Learning experiences after using the YouTube platform to deliver preliminary video lectures in a flipped classroom were assessed by 69 second-year students (52 males, 17 females) at Dankook University College of Dentistry, Korea, who attended periodontology lectures during 2 consecutive semesters of the 2016 academic year. The instructor uploaded the lecture videos to YouTube before each class. At the end of the second semester, the students were surveyed using a questionnaire devised by the authors.

**Results:**

Of the students, 53 (76.8%) always watched the lecture before the class, 48 (69.6%) used their smartphones, and 66 (95.7%) stated that they watched the lectures at home. The majority of the students replied that the video lectures were easier to understand than face to face lectures (82.6%) and that they would like to view the videos again after graduation (73.9%).

**Conclusion:**

Our results indicate that YouTube is an applicable platform to deliver video lectures and to expose students to increased learning opportunities.

## Introduction

Dental education has undergone unprecedented changes to more directly involve students in learning by focusing on critical thinking, problem-solving skills, and student-centered learning [1]. These innovations became possible due to refinements in information and communication technology and the increased prevalence of e-learning or online learning platforms [2]. One result has been the emergence of the ‘flipped classroom’ model as an alternative to conventional one-to-many lecture-based teaching. The flipped classroom is a teaching method that promotes the learning of basic concepts through pre-learning and concentrates on in-depth learning activities in class through actual problem-solving and clinical exposure. It is a type of blended learning in which in-class learning is integrated with online learning experiences, and students watch pre-recorded lecture videos prior to attending the class [3]. It is critical to provide a flexible environment that is free from time and space constraints to operate a flipped classroom successfully [4]. With this background, we aimed to confirm the applicability of YouTube as a delivery platform of micro-lecture videos to provide the flexible learning environment that is necessary for the flipped classroom.

## Case presentation

### Ethics statement

This study was approved by the Institutional Review Board of Dankook University Dental Hospital after receiving informed consent from the subjects (IRB approval no., DKU DH 2017-10-002).

### Case

**Fig. 1.**
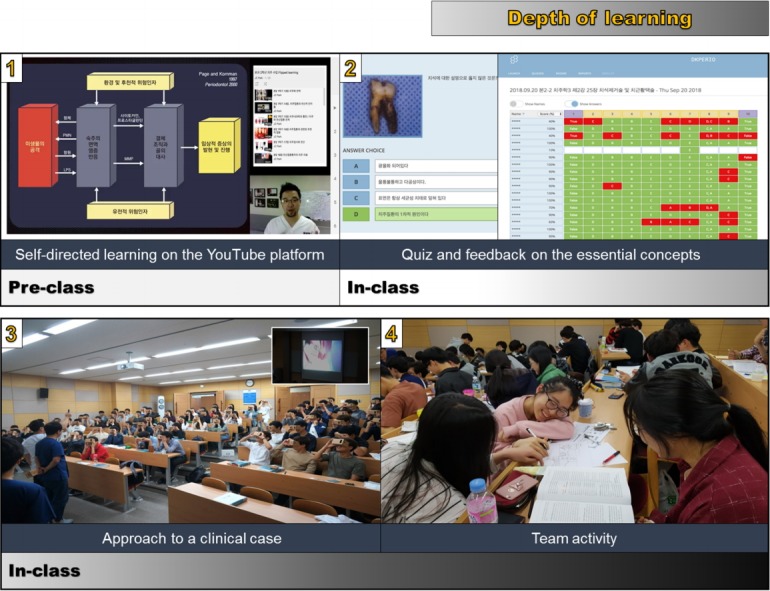
Implementation of the flipped classroom.

The participants were 69 second-year students of Dankook University College of Dentistry who attended a clinical periodontology course (52 males, 17 females). The periodontology course ran from March 2 to December 16, 2016. This course contained 1-hour weekly sessions, comprising a total of 14 hours. The instructor recorded the lecture videos using the Screencast application, which records the content of the presentations and the comments of the lecturer in real-time (Camtasia; TechSmith Corp., Okemos, MI, USA). The lecture videos were uploaded to YouTube before the class (https://www.youtube.com/jcparkland), as shown in Table 1 (Supplement 1). On average, the micro-lecture videos did not exceed 20 minutes in consideration of the students’ ability to focus. Abstracts of all micro-lectures were also uploaded as PDF files. The links to the You-Tube videos and the PDF files were posted together using G Suite for Education (Google, Mountain View, CA, USA). The students completed pre-learning by watching micro-lecture videos on the YouTube platform and reading the PDF abstract before attending class, and their understanding of the content of the micro-lecture videos was assessed through an in-class quiz related to the pre-learning content. Students’ answers to the quiz were shared in real time on the screen using the smartphone application Socrative (http://socrative.com) [5], which is a cloud-based student response system. For topics for which students’ understanding was considered to be lacking, immediate feedback was provided by introducing major concepts. Through such a process, students could thoroughly learn the basic concepts that they needed to be aware of. After checking and complementing the pre-learning process through the quiz and feedback, theoretical perspectives and clinical practice were integrated by linking the content with treatment/surgery fields through Google Cardboard virtual reality (VR) and live surgery broadcasting. This process enhanced the acquisition of actual knowledge and promoted motivation to learn by helping students indirectly experience how the theories they learned were applied in clinical settings. Moreover, after the process, in-depth learning of corresponding topics was promoted through various team-based learning activities, such as debates, and projects. The details of the pre-class and in-class process are shown in Fig. 1.

**Fig. 2.**
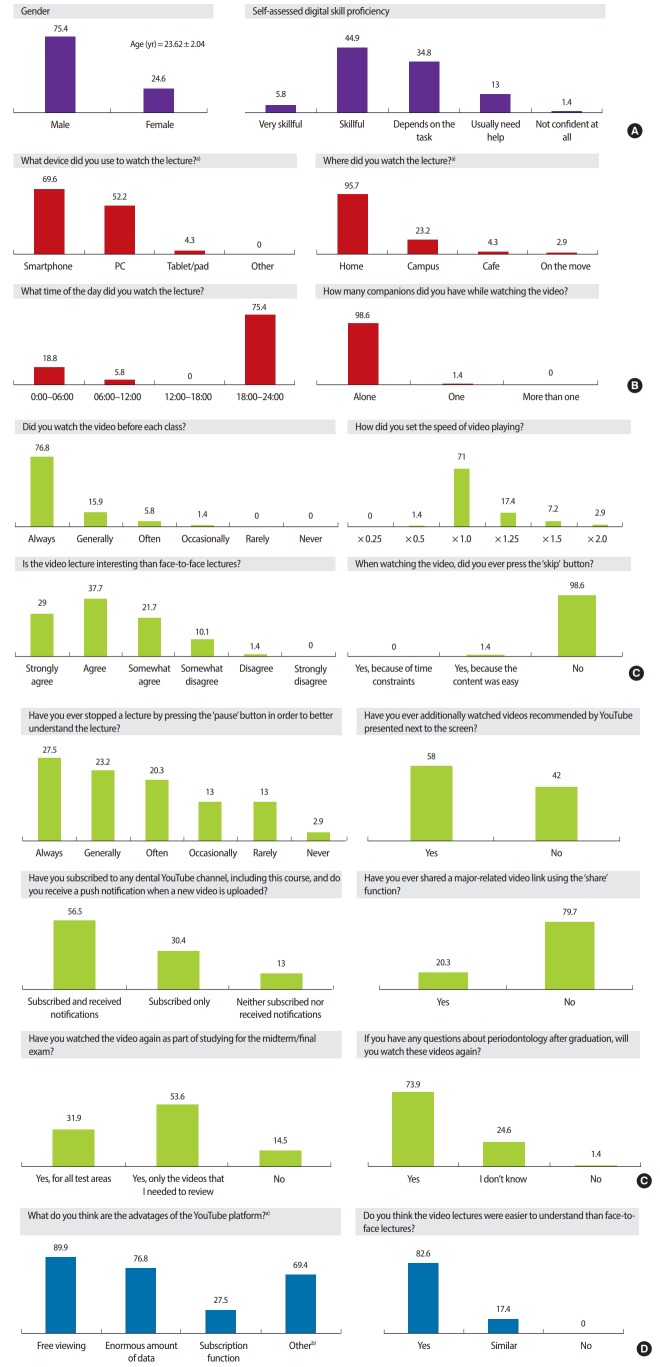
Descriptive statistics of the post-course survey items focusing on the learning experience using the YouTube platform. Values are presented as % or mean ± standard deviation. (A) Basic information. (B) Pre-learning environment. (C) Lecture viewing: learning attitude and awareness. (D) Assessment of the YouTube platform. PC, personal computer. ^a)^Multiple selection; ^b)^Sharing, virtual reality content, replay, feedback using comments, subtitles, and live streaming.

At the end of the second semester, the students were surveyed using the questionnaire devised by the authors. We developed a survey questionnaire to assess the students’ learning attitudes and the applicability of YouTube with a rubric, according to which each item was scored on a 6-point Likert scale. The questionnaire was developed by the researchers in advance and completed by consulting with 2 medical education experts. Students responded to the survey after the final examination using Google Forms with their smartphones in January 2017. We conducted a descriptive statistical analysis, including the frequency and percentage of answers. The raw data of this study are presented in Supplement 2. The major results were as follows (Fig. 2).

First, students usually watched the micro-lecture videos using smartphones (69.6%), at their home (95.7%) in the evening after school (75.4%), and they usually watched them alone (98.6%).

Second, almost all students came to class after watching the micro-lecture videos (92.7%), and they watched them at a speed within the normal range (range, × 1.0 to × 1.25; 88.4%), without using the skip function (98.6%), despite concerns. Sixty-seven students (97.1%) reported engaging in supplementary learning practices, such as returning to the previous stage after stopping the micro-lecture videos using the pause function of YouTube when they encountered difficulty in understanding while watching the lecture. In addition, many students reviewed the related micro-lecture videos to prepare for examinations (85.5%).

Third, 57 students preferred the micro-lecture videos because they perceived them to be more helpful for understanding class topics than offline lectures (82.6%), and 73.9% of the students reported that they intended to watch the videos again if they become curious about periodontology after graduation.

Fourth, regarding the functions of YouTube, 86.9% of the students subscribed to a YouTube channel related to their major, and as many as 58.0% of the students additionally watched the related videos that YouTube recommended, as well as the required micro-lecture videos their professor had uploaded. However, only 20.3% of the students reported having shared a video link with others.

Fifth, students stated that the YouTube platform had the advantage of allowing them to watch an enormous amount of data (76.8%) for free (89.9%), and YouTube functions such as VR content, replay, feedback using comments, subtitles, and live streaming helped their understanding.

## Discussion

A previous study reported that it was challenging to implement this novel teaching method due to technical difficulties, even though the students were interested and co-operative regarding the flipped classroom in dental colleges [6]. Our study has shown that the preclass lecture videos were well accepted by the students, and the students considered the YouTube platform to be an efficient platform to distribute the lecture videos. Many students thought that the video lectures were better than face-to-face lectures for understanding the content. The functions of the micro-lecture videos provided on the YouTube platform, such as pause, replay, speed adjustment, and comments, promoted students’ understanding by enabling iterative learning and two-way feedback. Furthermore, YouTube’s functions of sharing and exposing students to related videos helped students grow as subjects of learning by enabling students to experience more meaningful and personalized learning. Finally, the goal of these video lectures was to introduce the basic concepts of periodontology to the students, but we also intended to provide them learning materials suitable for later reuse whenever they wish to refresh their memory [7]. The majority of students said that they would watch the videos again, which is a very inspiring response, suggesting that this modality may be a useful means of continuing education in the near future. Additionally, since the YouTube dashboard contains suggestions of related videos, the students can freely peruse those related videos, eventually allowing them to access more learning materials and possibly cultivate further interest in certain topics. Most of all, it is noteworthy that students watched the micro-lecture videos at their own speed, using the functions of YouTube appropriately, with a sincere attitude, in convenient locations at any time they wanted. This fact implies that the YouTube platform could support the self-directed learning of students by providing high accessibility and serving as a tool that is fit for their needs.

As such, we suggest, based on our experiences, that YouTube is a suitable platform that facilitates self-directed learning of students, providing flexible and diverse learning forms for a flipped classroom. However, as this study involved a single dental school, it should be kept in mind that perceptions of efficacy may vary according to learners’ characteristics. Therefore, it is expected that research on various platforms for learners will continue in order to build more effective learning environments, and the flipped classroom will eventually be successfully integrated into dental education through suitable class design.

## Figures and Tables

**Table 1.** The list of lecture videos uploaded to YouTube over 2 semesters from March to December 2016

Topic	Duration (min:sec)	Direct link
1. Periodontal instruments	23:26	https://goo.gl/Md1aWi
2. Periodontal instrumentation	17:55	https://goo.gl/D6oKXB
3. Periodontal healing	15:00	https://goo.gl/1bOhYv
4. Mechanical plaque control	12:59	https://goo.gl/i5h8eb
5. Scaling and root planing	16:10	https://goo.gl/GNGE1K
6. Chemical plaque control	13:42	https://goo.gl/8ixJxZ
7. Principles of surgical treatment	14:56	https://goo.gl/UBRlrH
8. Emergency treatment	12:47	https://goo.gl/AdWb26
9. Subgingival curettage	17:17	https://goo.gl/0fZTBJ
10. Gingivectomy and gingivoplasty	16:20	https://goo.gl/vdj2h4
11. Flap operation	28:57	https://goo.gl/vkODnY
12. Guided tissue regeneration	18:52	https://goo.gl/vXCzVd
Average running time ± standard deviation	17:22±4:41	

## Supplementary material

Supplement 1. Sample video file : Lecture no. 10. Gingivectomy and Gingivoplasty.

Supplement 2. Data files are available from https://doi.org/10.7910/DVN/LTDX5Q.

Supplement 3. Audio recording of the abstract.
